# Medical exemptions to mandatory vaccinations: The state of play in Australia and a pressure point to watch

**DOI:** 10.1017/S0950268824000268

**Published:** 2024-02-22

**Authors:** William Kouji Yap, Katie Attwell

**Affiliations:** 1 UWA Medical School, The University of Western Australia, Perth, WA, Australia; 2 Wesfarmers Centre of Vaccines and Infectious Diseases, Telethon Kids Institute, Perth, WA, Australia; 3 School of Social Sciences, The University of Western Australia, Perth, WA, Australia

**Keywords:** public health, vaccination (immunization), COVID-19, health policy

## Abstract

Australia’s mandatory vaccination policies have historically allowed for non-medical exemptions (NMEs), but this changed in 2016 when the Federal Government discontinued NMEs for childhood vaccination requirements. Australian states introduced further mandatory vaccination policies during the COVID-19 pandemic for a range of occupations including healthcare workers (HCWs). There is global evidence to suggest that medical exemptions (MEs) increase following the discontinuation of NMEs; the new swathe of COVID-19 mandatory vaccination policies likely also placed further pressure on ME systems in many jurisdictions. This paper examines the state of play of mandatory vaccination and ME policies in Australia by outlining the structure and operation of these policies for childhood vaccines, then for COVID-19, with a case study of HCW mandates. Next, the paper explores HCWs’ experiences in providing vaccine exemptions to patients (and MEs in particular). Finally, the paper synthesizes existing literature and reflects on the challenges of MEs as a pressure point for people who do not want to vaccinate and for the clinicians who care for them, proposing areas for future research and action.

## Introduction

Australia is known for its strict vaccination policies. In 2016, the Federal Government discontinued non-medical exemptions (NMEs) (also known as Conscientious Objections) for childhood vaccination requirements. States also began introducing vaccine requirements for early education and care with no NMEs in the same year. Subsequently, Australian states initiated mandatory COVID-19 vaccination policies for a range of occupations including healthcare workers (HCWs) [1–8] during the pandemic. These policies did not permit NMEs and required vaccination of a large percentage of the Australian adult population concurrently [2–8]. The discontinuation or non-availability of NMEs presents a possible pressure point regarding provision of medical exemptions (MEs): vaccine refusers may flock to MEs instead [1, 5, 9–12].

With the tightening of exemptions and an increase in the number of mandatory vaccination policies, we could expect an increase in the demand for MEs in an environment in which exemptions are increasingly difficult to access. This may lead to stress on the ME system through attempts by parents, workers, citizens, and even some medical providers to manipulate that system. To prevent these downstream effects and to ensure that ME policies are implemented judiciously and effectively, it will be important to examine stakeholders’ and the public’s sentiments regarding vaccination MEs and the effects of these policies on the HCWs providing these MEs. Ahead of such empirical work, this paper conducts three important tasks. First, it outlines the structure and operation of mandatory vaccination and exemption policies in Australia for childhood vaccines, then for COVID-19, with a case study of the HCW mandates that were initiated in all Australian states early in the COVID-19 vaccine rollout. Next, the paper reviews the global literature regarding HCWs’ experiences in providing vaccine exemptions (and MEs in particular), considering implications for COVID-19 vaccine mandates. Finally, the paper outlines areas for future research and action regarding MEs in a context of heightened demand.

## Mandatory childhood vaccination policies in Australia

Australia’s National Immunization Program funds immunizations from birth through to adulthood. Federal ‘No Jab, No Pay’ and state-level ‘No Jab, No Play’ policies mandate most childhood vaccines. These mandates were introduced in their general current form from 2016 and onwards and, if a child is not fully vaccinated, restrict families from accessing federal financial support, or enrolling in childcare (in some states), respectively. NMEs, excepting special cases, are not accepted [1, 13]. However, MEs have been available under federal policy since 1997 to recognize children medically ineligible for vaccination as compliant [14].

The Federal ‘No Jab, No Pay’ Policy mandates vaccination for receipt of full Family Tax Benefit (FTB) Part A and childcare fee assistance. It outlines that one must ‘have an approved medical exemption recorded on the Australian Immunization Register (AIR)’ in order to qualify for a ME [13]. State-level ‘No Jab, No Play’ policies require children to be fully vaccinated to attend childcare and early education facilities. These policies span five Australian states, including New South Wales (NSW), Victoria, Queensland, Western Australia (WA), and South Australia (SA), outlined in Table 1. For each of these states, the federal ME form and process enable enrolment for children who cannot be vaccinated for medical reasons. Australia’s other states and territories (Tasmania, the Northern Territory and the Australian Capital Territory) do not mandate childhood vaccination for enrolment in childcare and early education facilities [1].

**Table 1.** Australia’s state-based ‘No Jab, No Play’ policies

	NSW	Victoria	Queensland	WA	SA
Date policy came into force	01/01/2014	01/01/2016	01/01/2016	22/07/2019	07/08/2020
Exemptions permitted	Medical + 4 special circumstances	Medical + 6 special circumstances	Medical	Medical + 7 special circumstances	Medical + 5 exceptions granted by state chief medical officer (not legislated)

## Australian HCW COVID-19 vaccine mandate policies

As demonstrated in the previous section, most Australian states have a history of mandating vaccines for childhood populations, making use of federal infrastructure for proof of vaccination and MEs. In addition, some Australian states mandate or previously mandated annual influenza vaccines for healthcare and/or aged care workers, as well as requiring proof of vaccination or immunity for commencement of employment for some diseases [5, 10–12, 15]. State governments therefore possessed the infrastructure and policy experience enabling them to promptly introduce COVID-19 mandates into populations that were familiar with – and broadly supportive of – previous mandates [1, 16].

Australia’s Constitution determines whether a particular policy area is a state or federal concern. The Australian Government possesses limited scope to directly impose public health controls, so their only COVID-19 vaccine mandate covered entry to the country from overseas [17–19]. However, states and territories used their more extensive powers to require vaccinations for entry from other states or countries, for particular types of employment, and for access to public spaces of entertainment, hospitality, and care [1].

All states and territories imposed vaccine mandates for COVID-19 for HCWs at some point, but most have since been revoked or have lapsed [2–8]. Table 2 demonstrates: 1) how some of the earliest mandates unfolded and functioned for a population that was at highest risk of transmission and transmitting the virus themselves and 2) differences between state approaches, including the *timing*, the type of enabling *legislation*, and whether, when, and how the mandates were *rescinded*) (Table 2). The third point of difference between the states – ME processes and proof – is examined in the next section.

**Table 2.** COVID-19 mandatory vaccination policies for HCWs in Australia

State	WA [8, 19]	NSW [4, 15, 20]	Victoria [3, 21]	Queensland [2, 22]	Tasmania [23, 24]	SA [7, 25]	Northern Territory [5, 26]	Australian Capital Territory [6, 27]
Policy Name	*COVID-19 Mandatory Vaccination – Employee Restrictions on Access to Health Care Facilities – Guidelines* (Guidelines)	*Public Health (COVID-19 Vaccination of Health Care Workers) Order 2021*	*Pandemic COVID-19 Mandatory Vaccination (Specified Facilities) Order No. 1.*	*COVID-19 Testing and Vaccination Requirements (Contact by Health Workers with Cases) Direction*	*Public Health Act 1997* *DIRECTION UNDER SECTION 16* *(Mandatory Vaccination of Certain Workers – No. 3)*	*Emergency Management (Healthcare Setting Workers Vaccination) (COVID-19) Direction 2021*	*COVID-19 Directions (No. 55) 2021: Directions for mandatory vaccination of workers to attend the workplace*	*Public Health (Health Care and Support Workers COVID-19 Vaccination) Emergency Direction 2021*
Date enacted for first HCWs	16/09/2021	29/09/2021	15/12/2021	31/03/2021	31/10/2021	01/11/2021	13/10/2021	15/10/2021
Enacted under	*Public Health Act 2016 (WA)*	*Public Health Act 2010 (NSW)* 22/08/2023	*Public Health and Wellbeing Act 2008 (VIC)*	*Public Health Act 2005 (QLD)*	*Public Health Act 1997*	*Emergency Management Act 2004* (SA)	*Public and Environmental Health Act 2011 (NT)*	Public Health Act 1997 (ACT)
Date ended and process	05/04/2023: *COVID-19 Mandatory Vaccination and Vaccination Program Policy*	Superseded by *Occupational Assessment, Screening, and Vaccination Against Specified Infectious Diseases Policy*	Not rescinded: superseded by Secretary Directions 13/10/2022	02/09/2022: Revoked by *Revocation of Workers in healthcare setting (COVID-19 Vaccination Requirements)* Direction (No. 4)	30/06/2022 Latest rendition: *Public Health Act 1997 DIRECTION UNDER SECTION 16 (Mandatory Vaccination of Certain Workers – No. 12)* lapsed.	14/10/2022: *Emergency Management (Healthcare Setting Workers Vaccination No. 7*) was ceased	22/4/2022: Revoked by *Public and Environmental Health Act 2011 COVID-19 Directions (No. 53) 2022: Revocation of COVID-19 Directions (No. 55) 2021*	13/05/2022: Revoked by *Public Health (Health Care and Support Workers COVID-19 Vaccination) Emergency Direction Revocation 2022*

Of note, most states invoked Public Health Acts and enacted the mandates close to the end of 2021, often distinguishing between categories of HCW (based on exposure and the vulnerability of patients in their care) and introducing requirements in a tiered fashion. Further, most states rescinded their policies during mid-2022, by withdrawing the public directions in place. However, NSW maintains mandatory vaccination through their *Occupational Assessment, Screening, and Vaccination Against Specified Infectious Diseases Policy*, as does Victoria, through invoking Secretary Directions [9, 21].

## Medical exemption policies in Australia: Attaining an exemption

The COVID-19 and childhood mandatory vaccination policies described above utilize the same general methods for MEs. To obtain a ME, an individual must be certified by an eligible practitioner (certain GPs and specialists) as having a medical contraindication, or (for some diseases) possessing natural immunity. In some additional special cases, an individual will be deemed to meet immunization requirements without requiring a ME. These include: taking part in an approved vaccine study, the vaccine being temporarily unavailable, the individual having been vaccinated overseas, or the Secretary (a senior official in the Department of Health) determining that the individual meets the requirements [28, 29].

To record a ME for official purposes, an eligible practitioner must certify either medical contraindication or natural immunity through the AIR site or submit an AIR medical exemption form [30]. Medical contraindications are ‘permanent’ or ‘temporary’. Permanent contraindications recognized by the AIR are if an individual: ‘had anaphylaxis after a previous dose of a vaccine’, ‘had anaphylaxis after a dose of any component of a vaccine’, or ‘are significantly immunocompromised’ (for live attenuated vaccines only). Grounds for a temporary vaccine exemption are: ‘acute major medical illness’, ‘significant immunocompromise of short duration (live attenuated vaccines only)’ or ‘the individual is pregnant (live attenuated vaccines only)’. If they ‘have natural immunity’ (for hepatitis B, measles, mumps, rubella and chickenpox only), they are also eligible for ME. Natural immunity is assessed via laboratory testing or physician-based clinical diagnosis [28, 31, 32].

## Australian COVID-19 exemption policies

All states permitted both temporary and permanent MEs for COVID-19 vaccinations, requiring medical professionals to complete the AIR’s Immunization Medical Exemption Form and upload it to the register [31]. For both temporary and permanent exemptions, the federal system only acknowledges a blanket COVID-19 vaccine exemption for individuals with a medical contraindication to all available brands of COVID-19 vaccine [30, 33]. Some states’ mandates also require(d) their own state-based documentation, or enabled applicants to just apply for a state-based exemption. Table 3 details the processes for obtaining a COVID-19 ME in different states.

**Table 3.** Australian states’ COVID-19 medical exemption policies

State	AIR and/or state requirements	Further details
WA [34, 35]	AIR form	Temporary exemptions (processed by Chief Health Officer) can be applied for whilst awaiting an exemption on AIR.
NSW [34]	AIR exemption form OR NSW Health’s *COVID-19 Vaccine Medical Contraindication Form*	If HCW completes NSW Health’s form, they should provide a copy to the patient to certify exemption
Victoria [34, 36]	Both the AIR exemption form AND Victoria’s *Medical Exemption to COVID-19 Vaccination Guidance*	AIR certificate used as proof of exemption
Queensland [34, 37, 38]	AIR exemption form	Other exemptions (e.g., participation in COVID-19 vaccine trial) can be documented in a clinical document, accounting for the patient’s medical history, ATAGI guidelines, and Queensland Health guidelines.
Tasmania [23, 34]	Both the AIR exemption form AND Tasmania’s *COVID-19 Vaccine Medical Contraindication Form*	AIR certificate used as proof of exemption
SA [34]	Both the AIR exemption form AND SA Health’s *COVID-19 Vaccine Medical Exemption Application Form*	The state Chief Public Health Officer or delegate must approve every exemption.
NT [34]	Either the AIR exemption form OR a medical certificate	The medical certificate must state all available COVID-19 vaccines are medically contraindicated, in accordance with ATAGI guidelines
ACT [34]	Both the AIR exemption form AND the ACT’s *COVID-19 Vaccine Medical Contraindication or Temporary Exemption Form*	A copy of the ACT form should be provided to the patient as proof of exemption

The ‘double requirement’ in some states and territories for local and national forms has been attributed to policymakers’ desires to protect the system and the HCWs granting exemptions. Victoria’s Acting Chief Health Officer, Professor Ben Cowie, commented that his government’s requirement of both a formal AIR certificate and a state-level guidance form for a valid exemption, aimed to ‘provide GPs with a really tight and clear process that they can work through with their patients’ [34]. In SA, the Chief Public Health Officer or her delegate must formally approve an exemption. The RACGP SA and NT Chair, Dr. Daniel Byrne, anticipated ‘we won’t get the arguments and pushback that seem to be happening interstate’ [34].

## Medical exemptions: The pressure point

As evidenced above, policymakers have been cognizant of how COVID-19 exemption policies need to be conceptualized meticulously. However, another potential issue is the sheer volume of applications that the system might have to deal with. Studies in various global contexts indicate that following the discontinuation of NMEs to mandatory childhood vaccination policies, there has been a corresponding increase in demand for MEs.

Yap et al. examined the experiences of Specialist Immunization Clinic (SIC) Providers in Australia following the introduction of the Federal ‘No Jab, No Pay’ policy [39]. SICs are referral-only services for patients who require specialist immunization advice and care [39]. SIC providers reported an increase in the demand for MEs following the policy change, and the mean number of immunizations per clinic per month increased from 3.1 to 5.1. Further, in California, Mohanty et al. studied the discontinuation of NMEs from 2015 [40]. They found that although overall numbers remained low, MEs increased 250% (from 0.2% in 2015–2016 to 0.7% in 2017–2018), and that the counties that previously held the highest rates of personal belief exemption (a type of NME) had the largest increases in rates of MEs. Gromis and Liu investigated spatial clustering of MEs following California’s policy change, finding clusters for MEs in similar geographic regions to previous clusters of NMEs [41]. These findings suggest an association between previously high NME rates and increasing ME rates, which may indicate an influx of people seeking MEs when unable to obtain NMEs.

However, data from the National Centre for Immunization Research and Surveillance (NCIRS) 2017 Annual Immunization Coverage Report show the opposite effect of Australia’s policy change [42]. The NCIRS data are to do with children aged 6 months to 10 years with at least one new vaccination exemption due to a medical contraindication recorded on the AIR between 2011 and 2017. Between 2011 to 2015, there was an increasing number of new MEs being granted, with new MEs more than doubling between 2014 and 2015 (635 to 1,401). However, new MEs *decreased* dramatically in 2016 and 2017 following the elimination of NMEs (from 1,401 in 2015 to 508 in 2017). NCIRS linked the drop to stricter granting criteria and conditions from 2016, aligning with the ‘No Jab, No Pay’ policy [42, 43]. However, data on exemptions held by the Federal Government have not been analysed since this time and are not available publicly.

Meanwhile, as described above, the COVID-19 pandemic saw new mandatory vaccination policies across Australian states. There are no public data on the number of MEs requested or recorded on the AIR during the pandemic for any type of COVID-19 vaccine. According to 9 News (an Australian media company), as of 17 October 2021, 156 permanent contraindications and 1,071 temporary contraindications were submitted by doctors to the AIR [44]. There are no more recent figures available in the public domain. However, there is no doubt that the availability of both temporary and permanent exemptions throughout the pandemic led to further pressure on providers to grant MEs to people who did not want to be vaccinated against COVID-19.

## Potential implications for the rising demand for exemptions

Whilst the evidence of a replacement effect for childhood NMEs with MEs is mixed [45], mandatory COVID-19 vaccination policies generated significant demand for MEs. In light of the issues discussed above, this section examines the potential problems that an increasing demand for MEs might create.

### Stressful and hostile consultations

Literature shows that vaccine providers find discussions with hesitant or resistant parents and consumers stressful even when NMEs are available. Berry et al.’s qualitative study – undertaken prior to Australia’s removal of NMEs at a federal level – uncovered that most GPs and nurses found vaccine objection consultations challenging. They saw vaccine objection as a challenge to their legitimacy as medical experts, generating conflicts between their professional duties and contractual obligations, and presenting communication roadblocks [46]. Following the policy change, the study of SIC providers in Australia found that 69% (*n* = 11) of the providers surveyed felt that Australia’s ‘No Jab’ childhood vaccination policies were moderately or seriously stressful in their practice. Respondents reported encountering hostility during their interactions with patients, including threats and even physical violence [39]. Echoing this, during the pandemic, Dr. Karen Price, president of RACGP, described ‘aggressive and abusive’ behaviour towards doctors by patients who were trying to get out of COVID-19 vaccine requirements. These included threats demanding fraudulent ME paperwork, and cash bribes of up to thousands of dollars [34, 47, 48].

### Concerning medical practices

Given the stressful nature of consultations with exemption-seeking patients, HCWs may seek to mitigate pressures by granting questionable MEs [39, 40, 46]. Yap et al. reported that 88% (*n* = 14) of Australian SIC respondents felt pressured to exempt ineligible children, [39] while Salmon et al. found that up to a quarter of providers in four US states would grant MEs in the absence of valid medical contraindications [46]. Doctors in the Australian state of Victoria were found to be granting fraudulent exemptions after the introduction of its ‘No Jab, No Play’ policy in 2016. In response, the Victorian Government amended the policy in 2017 [49], revoking parents’ ability to obtain a letter of medical contraindication from their doctor and recognizing only MEs lodged upon the AIR. HCWs face consequences for inappropriate exemptions: the Australian Health Practitioner Regulation Agency (AHPRA) stated during the COVID-19 pandemic, that if an exemption is found to be unwarranted, the consequences for the practitioner could be ‘significant’. HCWs may therefore feel stuck between their obligations to patients and their obligations to regulatory organizations, which RACGP President Dr. Price said ‘does add to our stress levels’ [34, 47]. Another response may also be to outright refuse to see patients seeking medical exemptions, which some physicians in the United States have been doing for years in response to parents seeking either medical or NMEs [39, 50].

## Conclusion

The landscape of childhood and COVID-19 mandatory vaccination and exemption policies is evolving, but vaccine mandates appear to be an enduring policy response for governments in both business as usual and crisis scenarios globally. The resultant increasing demand for MEs will place mounting pressure upon HCWs as ‘frontline’ workers in compliance and enforcement processes. Thwarted vaccine refusers may direct their frustration at HCWs as the ‘gatekeepers’ of medical exemptions [41]. Governments should funnel resources towards ME policies to ensure that they are tightly regulated, maximizing compliance and minimizing exploitation. Strategies could be implemented at various levels to support HCWs, whilst not undermining the concerns and autonomy of patients. At the policy level, as in the Australian state of South Australia, requiring the approval of a senior health bureaucrat may take pressure off frontline providers [34]. At the clinic level, as noted by Yap et al., allowing for 2-clinician consultations may be helpful [39]. Finally, at the individual clinician level, training in strategies such as 1) exploring and informing 2) mobilizing clinical rapport 3) first doing no harm to the clinical relationship, as described by Berry et al. [48], may enable vaccine providers to better handle stressful interactions with exemption-seekers. Further empirical research into COVID-19 mandatory vaccination and exemption policies, including HCW experiences of exemption-seeking, can advance our understanding and the implementation of current and future policies in the diverse range of countries that employ vaccine mandates.

## Data Availability

This document was prepared by searching through public policy documents, journal articles, and webpages with the exception of the Tasmanian public health policy documents. These documents can be provided upon request.
